# Classification and Design of HIV-1 Integrase Inhibitors Based on Machine Learning

**DOI:** 10.1155/2021/5559338

**Published:** 2021-04-01

**Authors:** Junlin Zhou, Juan Hao, Lianxin Peng, Huaichuan Duan, Qing Luo, Hailian Yan, Hua Wan, Yichen Hu, Li Liang, Zhenjian Xie, Wei Liu, Gang Zhao, Jianping Hu

**Affiliations:** ^1^Key Laboratory of Coarse Cereal Processing, Ministry of Agriculture and Rural Affairs, School of Pharmacy, Key Laboratory of Medicinal and Edible Plants Resources Development of Sichuan Education Department, Sichuan Industrial Institute of Antibiotics, Chengdu University, Chengdu 610106, China; ^2^College of Mathematics and Informatics, South China Agricultural University, Guangzhou 510106, China

## Abstract

A key enzyme in human immunodeficiency virus type 1 (HIV-1) life cycle, integrase (IN) aids the integration of viral DNA into the host DNA, which has become an ideal target for the development of anti-HIV drugs. A total of 1785 potential HIV-1 IN inhibitors were collected from the databases of ChEMBL, Binding Database, DrugBank, and PubMed, as well as from 40 references. The database was divided into the training set and test set by random sampling. By exploring the correlation between molecular descriptors and inhibitory activity, it is found that the classification and specific activity data of inhibitors can be more accurately predicted by the combination of molecular descriptors and molecular fingerprints. The calculation of molecular fingerprint descriptor provides the additional substructure information to improve the prediction ability. Based on the training set, two machine learning methods, the recursive partition (RP) and naive Bayes (NB) models, were used to build the classifiers of HIV-1 IN inhibitors. Through the test set verification, the RP technique accurately predicted 82.5% inhibitors and 86.3% noninhibitors. The NB model predicted 88.3% inhibitors and 87.2% noninhibitors with correlation coefficient of 85.2%. The results show that the prediction performance of NB model is slightly better than that of RP, and the key molecular segments are also obtained. Additionally, CoMFA and CoMSIA models with good activity prediction ability both were constructed by exploring the structure-activity relationship, which is helpful for the design and optimization of HIV-1 IN inhibitors.

## 1. Introduction

Acquired immune deficiency syndrome (AIDS) is a systemic immune dysfunction syndrome caused by the infection of human immunodeficiency virus (HIV) infection, inducing the destruction of CD4^+^ T lymphocytes [1–3]. HIV can be divided into two subtypes: HIV-1 (i.e., the main pathogen of AIDS) and HIV-2. HIV-1 is characterized by strong infection, rapid mutation, and high mortality and can be transmitted through blood, mother-infant, sexual intercourse, etc. [4–8]. Since the first case of HIV-1 infection in 1981, the number of AIDS patients has exploded worldwide [9]. According to World Health Organization (WHO) data of 2019, more than 38 million people have been infected, and 7.1 million of them have died [10]. Highly active antiretroviral therapy (HAART) is the main strategy in the clinical treatment of AIDS—the combination of drugs inhibiting both reverse transcriptase (RT) and the protease (PR), which can reduce the damage of virus to immune system [11]. However, the high variability of HIV-1 results in poor efficacy of HAART treatment, leading to the emergence of drug-resistant virus strains. It is urgent to identify new targets and develop novel structural inhibitors [12–14].

As such an attractive and important target, HIV-1 integrase (IN) is an essential enzyme in the HIV-1 lifecycle responsible for inserting the reverse-transcribed viral genome into the host DNA through 3′ processing (3′-P) and strand transfer (ST) reaction [15, 16]. Unlike PR and RT, there is neither known functional analog of IN in human cells nor apparent cellular toxicity for IN inhibitors [17, 18]. Encoded by the pol gene, HIV-1 IN is composed of 288 residues with molecular weight of 32 kDa, which can be divided into three domains: N-terminal domain (NTD, residues 1-49), catalytic core domain (CCD, residues 50-212), and C-terminal domain (CTD, residues 213-288) [19]. The zinc finger in NTD is conductive to the stability of the whole IN enzyme; proper chelation of DDE motif (i.e., Asp64, Asp116, and Glu152) in CCD with two Mg2^+^ ions is essential to maintain high enzymatic activity; CTD serves as the nonspecific binding to viral DNA [20–26].

There are ten main types of HIV-1 IN inhibitors currently reported: diketoacids, diazonaphthalene derivatives, quinolone acids, pyrimidine ketone, sulfur nitrogen thiozapine, polyhydroxy arylcyclic compounds, disulfoxide compounds, benzene sulfonamides, coumarin derivatives, salicylhydrazide derivatives, etc. [27–33]. Diketoacids are the most fully studied and most promising inhibitors against HIV-1 IN, showing high efficiency, high selectivity, and low toxicity [34–36]. In terms of inhibitory mechanism of diketoacid compounds, the carbonyl and carboxyl groups both are, respectively, chelated with two different Mg2^+^ ions, which significantly weakens ST reaction by destroying metal-DDE recognition. Raltegravir (RLT) was the first approved IN inhibitor drug through FDA in 2007, followed by elvitegravir (EVG) and dolutegravir (DTG) for clinical use [37–39]. Here, all three belong to diketoacid compounds.

A lot of experimental and theoretical studies have involved IN-ligand recognition, inhibition mechanism, and molecular modification of diketoacid compounds. Two important scientific problems remain unclear: (1) for many IN inhibitors reported, is there a good classification method to determine their activity? (2) How to effectively modify the diketoacid inhibitor by obtaining the key groups that affect molecular activity and then combining 3-dimensional quantitative structure-activity relationship (3D-QSAR) results? In this work, HIV-1 IN inhibitors were first collected to establish a personalized database; the activity prediction model and the key groups affecting the activity both were obtained with recursive partition (RP) and naive Bayes (NB) model; finally, based on the structure and activity data of pyruvic acid inhibitors against HIV-1 IN, a 3D-QSAR model with good predictive ability was proposed [40–42]. In particular, the quantitative relationship between molecular structure (such as spatial conformation, electrostatic characteristics, hydrophobicity, and H-bond) and its inhibitory activity was explored, which will provide theoretical guidance for the design of effective anti-AIDS drugs.

## 2. Methods

### 2.1. Preparation of HIV-1 IN Inhibitor Database

The established HIV-1 IN inhibitor database contains 682 inhibitors and 1103 noninhibitors (1785 molecules in total). All data on the structure and activity of small molecules were obtained from ChEMBL, Binding Database, DrugBank, PubMed, and 40 recent references. The IC_50_ value of 4600 *μ*m was set as the criterion for defining an inhibitor. In data processing, 1 and 0 were adopted to characterize inhibitors and noninhibitors, respectively. All the small molecules were generated using ChemOffice package with Gasteiger-Hückel charge attached and then optimized by the steepest descent (1000 steps) and the conjugate gradient (1000 steps) algorithms based on Tripos force field of SYBYL package [43]. The convergence criterion is less than 4.182 kJ·mol^−1^·nm^−1^ for energy gradient. The optimized structure is the basis of the subsequent molecular descriptor and molecular fingerprint calculations.

### 2.2. Calculation of Molecular Descriptors and Molecular Fingerprints

Molecular descriptors and molecular fingerprints of personalized database elements both were calculated with Discovery Studio 3.5 (DS 3.5) package. Here, a total of 13 molecular descriptors widely used in ADME prediction were adopted for calculation: apparent partition coefficient (logD), octanol-water partitioning coefficient (AlogP), the number of rotatable bonds (*n*_rot_), molecular weight (MW), the number of H-bond donors (*n*_HBD_), the number of H-bond acceptors (*n*_HBA_), the sum of oxygen and nitrogen atoms (*n*_O+N_), polar surface area (PSA), the number of aromatic rings (*n*_AR_), the number of rings (*n*_*R*_), molecular solubility (logS), molecular fraction polar surface area (MFPSA), and molecular surface area (MSA) [44–46].

The SciTegic extended link fingerprints (i.e., FCFP, ECFP, and LCFP) and path-based ones (i.e., FPFP, EPFP, and LEFP) both were calculated using Morgan algorithm [47, 48]. The first letter of molecular fingerprint, F/E/L, respectively, represents atomic functional role code, the properties used in the Daylight atomic invariants rule, and atomic type code of AlogP. Atomic functional role code (i.e., letter F) mainly includes the combinations of H-bond acceptor, H-bond donor, positive ionization, negative ionization, and aromatic with halogen elements. Letter E consists of the sum of connection number among atoms, element types, atomic charge, atomic weight, etc. Letter L is used to characterize the 120 atomic types involved in AlogP calculation. The second letter, C/P, respectively, stands for extended-connection molecular fingerprint and path-based one. The third and fourth letters are derived from the initial capitalization of the “finger print” word. Four-letter molecular fingerprints are often followed by Arabic numerals 4 or 6, indicating the maximum distance between atoms. As an important complement to molecular descriptors for drug-like compounds, molecular fingerprint parameters have been widely used in the classification and prediction of inhibitors and noninhibitors.

### 2.3. Recursive Partitioning Classifiers

Recursive partitioning (RP) is a classification statistical method which can directly predict inhibitors and noninhibitors in the form of decision tree, on the basis of compound data processing and biological activity threshold criteria. Depth and node both are two important parameters of decision tree, respectively, corresponding to the complexity of the whole event and the judgment process [49–51]. For example, when a compound is at the logD node, its calculated data can be compared with the threshold value and partitioned and then recompared and repartitioned at the next RP node, until the RP is infeasible to continue at the bottom of tree. The criterion for stopping partitioning is that the classification effect cannot be improved or the remaining samples are too small. As far as the tree depth is concerned, the larger the value, the better the classification of the training data, despite the risk of overfitting; a smaller tree depth indicates that the accuracy of feature recognition in the training set is slightly lower, and the tree shows good adaptability to the new dataset; generally, the tree depth of 3 to 10 is a more appropriate. In accordance with the golden section ratio, the database was divided into the training set containing 1485 compounds and the test set containing 300 compounds. The decision tree was established based on the training set, and the accuracy of model prediction was evaluated from the test set data.

### 2.4. Naive Bayesian Classifiers

In addition to RP method, naive Bayesian (NB) model was also performed to develop classifiers to distinguish HIV-1 IN inhibitors from noninhibitors [52–55]. Firstly, the *f* vector (*f* = <*f*_1_, *f*_2_, ⋯, *f*_*n*_>) was set, and the component vectors (*f*_1_, *f*_2_,…and *f*_*n*_) were, respectively, calculated to obtain the eigenvectors (*F*_1_, *F*_2_,… and *F*_*n*_), which can be used to represent the corresponding molecular descriptors or molecular fingerprints. According to Bayes' theorem, the conditional probability and marginal probability of two events can be correlated, as shown in formula (1):

(1) $$\begin{array}{c} p\left(C\;|\;F_{1},F_{2},\cdots ,F_{n}\right)=\frac{p\left(C\right)p\left(F_{1},\cdots F_{n}\;|\;C\right)}{p\left(F_{1},\cdots ,F_{n}\right)}\;. \end{array}$$

Here, *C* stands for the classification of compounds; *p*(*C* | *F*_1_, *F*_2_, ⋯, *F*_*n*_) is the posteriori probability after classification; *p* (*C*) is the prior probability obtained from the training set; *p* (*F*_1_, ⋯, *F*_*n*_ |  *C*) is for the conditional probability of compounds having a specific molecular descriptor; *p* (*F*_1_, ⋯, *F*_*n*_) represents the marginal probability (or total probability) for the occurrence of all particular molecular descriptors. In NB model, each molecular descriptor is independent of each other, from which formula (2) can be obtained:

(2) $$\begin{array}{c} p\left(F_{1},\cdots ,F_{n}\;|\;C\right)=p\left(F_{1}\;|\;C\right)\cdots p\left(F_{n}\;|\;C\right)=\overset{n}{\underset{i=1}{\prod }}p\left(F_{i}\;|\;C\right). \end{array}$$

Based on the data of training set, all the coefficients required in formula (2) can be calculated by formulae (3) and (4):

(3) $$\begin{array}{c} p\left(F_{i}=f_{i}\;|\;+\right)=\frac{\text{count}\left(F_{i}=f_{i}\cap C=+\right)}{\text{count}\left(C=+\right)}, \end{array}$$

(4) $$\begin{array}{c} \;p\left(F_{i}=f_{i}\;|\;-\right)=\frac{\text{count}\left(F_{i}=f_{i}\cap C=-\right)}{\text{count}\left(C=-\right)}\;. \end{array}$$

In this work, all compounds are divided into either HIV-1 IN inhibitors or noninhibitors. *p* (+) and *p* (-), respectively, represent the prior probability grouped into inhibitors and noninhibitors. The posterior probability of the compound being an inhibitor (*p*) or a noninhibitor (*q*) is calculated as follows:

(5) $$\begin{array}{c} p=\frac{p\left(+\right)}{p\left(F_{1}=f_{1},\cdots ,F_{n}=f_{n}\right)}\overset{n}{\underset{i=1}{\prod }}p\left(F_{i}=f_{i}\;|\;+\right),\\ q=\frac{p\left(-\right)}{p\left(F_{1}=f_{1},\cdots ,F_{n}=f_{n}\right)}\overset{n}{\underset{i=1}{\prod }}p\left(F_{i}=f_{i}\;|\;-\right), \end{array}$$

where the marginal probability *p*(*F*_1_ = *f*_1_, ⋯, *F*_*n*_ = *f*_*n*_) is a constant, and the sum of *p* and *q* is equal to 1.

### 2.5. Evaluation of Classification Model Quality

The prediction ability of NB and RP models can be evaluated with lots of parameters, including true positives (TP), true negatives (TN), false positives (FP), false negatives (FN), sensitivity (SE), specificity (SP), prediction accuracy of TP (PRE1), prediction accuracy of TN (PRE2), and Matthews correlation coefficient (*C*). Their specific calculation formula is as follows:

(6) $$\begin{array}{c} \text{SE}=\frac{\text{TP}}{\text{TP}+\text{FN}}, \end{array}$$

(7) $$\begin{array}{c} \text{SP}=\frac{\text{TN}}{\text{TN}+\text{FP}}, \end{array}$$

(8) $$\begin{array}{c} \text{PRE}1=\frac{\text{TP}}{\text{TP}+\text{FP}}, \end{array}$$

(9) $$\begin{array}{c} \text{PRE}2=\frac{\text{TN}}{\text{TN}+\text{FN}}, \end{array}$$

(10) $$\begin{array}{c} C=\frac{\text{TP}\times \text{TN}-\text{FN}\times \text{FP}}{\sqrt{\left(\text{TP}+\text{FN}\right)\left(\text{TP}+\text{FP}\right)\left(\text{TN}+\text{FN}\right)\left(\text{TN}+\text{FP}\right)}}. \end{array}$$

### 2.6. Three-Dimensional Quantitative Structure Activity Relationship

The quinolinone acid compounds—a class of HIV-1 IN inhibitors—were randomly divided into a training set (18 molecules in total) and a test set (4 molecules in total), and their experimental IC_50_ values were transformed into negative logarithmic form (i.e., pIC_50_). According to the three-dimensional quantitative structure activity relationship (3D-QSAR) theory, molecules with similar conformations tend to have similar biological activity. In this work, compound # 2, which has been resolved and has good activity, is selected as the template molecule [56]. Before constructing the 3D-QSAR models, all molecules are aligned according to the principle that common substructures overlap each other, which is done in SYBYL-X1.3. In addition, comparative molecular similarity index analysis (CoMSIA) and comparative molecular field analysis (CoMFA) both are currently the two most widely used 3D-QSAR methods. In order to establish CoMSIA model, the superimposed inhibitors were placed in the spatial grid, and a series of sp^3^-hybridized probe particles (such as C^+^, CH_4_, H^+^, and H_2_O) were rolled to calculate the interactions between probe and inhibitor. Based on different spatial coordinates of probes, all the field data of inhibitors were obtained, including steric field (S), electrostatic field (E), hydrophobic field (HD), H-bond acceptor (A), and H-bond donor (D) [57–61]. Compared with CoMSIA model, CoMFA only provides information on S and E fields.

Partial least squares (PLS) method was used for regression analysis of the training set. Leave-one-out (LOO) method was adopted for cross validation to gain the optimal numbers of component (ONC) and determination coefficient *q*^2^. Based on the ONC values, 3D-QSAR models were established by noncross validation, and a series of parameters including correlation coefficient *r*^2^, estimated standard error Es, root mean square error (RMSE), and *F*-test values were obtained accordingly. These parameters can be used to evaluate the stability and predictive ability of the models and predict biological activity of the molecules in test set [62–64]. The prediction correlation coefficient for the test set is calculated by equation (11):

(11) $$\begin{array}{c} r_{p}^{2}=\frac{\text{SD}-\text{PRESS}}{\text{SD}}. \end{array}$$

In equation (11), SD represents the deviation-square sum between experimental biological activity data in the test set and the average biological activity in the training set, and PRESS indicates the error-square sum of the predictive biological activity in the test set with experimental biological activity.

## 3. Results and Discussion

### 3.1. Classification Based on Molecular Descriptors

**Figure 1:**
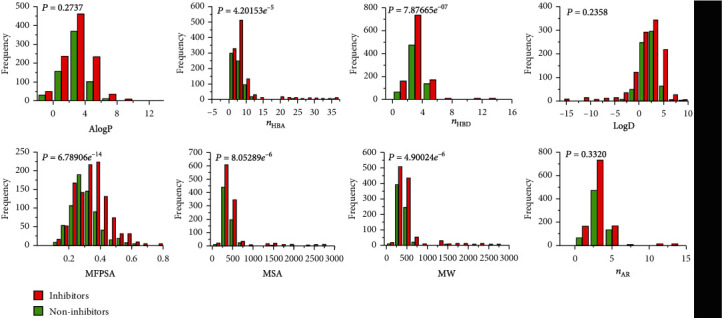
Distributions of eight molecular descriptors of both inhibitors and noninhibitors.

Compounds with similar biological activity usually have some similar molecular descriptors, such as reasonable hydrophilicity and H-bond number and volume. In theory, molecular descriptors can be partially used to classify inhibitors and noninhibitors.  Figure 1 shows the distributions of eight molecular descriptors (i.e., AlogP, logD, MW, *n*_HBA_, *n*_HBD_, MSA, *n*_AR_, and MFPSA). The distribution of AlogP ranged from -13.707 to 13.333, with an average of 2.523. Specifically, the average values for 682 inhibitors and 1103 noninhibitors were 2.608 and 2.470, respectively. Then, *t*-test was used to evaluate the significant difference of AlogP between inhibitors and noninhibitors. At 95% confidence level, the *P* value related to the difference between the two types of molecules was 0.274, indicating that there was no significant difference between the two distributions. Similarly, logD and *n*_AR_ both also have high *P* values of 0.236 and 0.332, respectively. Obviously, these three molecular descriptors with higher *P* values cannot be used to distinguish HIV-1 IN inhibitors from noninhibitors.

In addition, the *P* value of the other five molecular descriptors (i.e., MW, *n*_HBA_, *n*_HBD_, MSA, and MFPSA) were relatively small, with a minimum of 6.78*e*^−14^ and a maximum of 4.9*e*^−4^. Given the relatively scattered distribution and small overlap of these parameters, it is obvious that they cannot be used to accurately distinguish inhibitors from noninhibitors.

### 3.2. Classification Based on Recursive Partition Model

According to the above analysis, using a single molecular descriptor cannot classify compounds well. In order to establish a more accurate and understandable classification model, IC_50_ values were set as the classification basis and recursive partitioning (RP) model was adopted. In this model, molecules were divided into smaller and smaller subsets and finally presented in the form of decision tree. According to our experiments, the classification performance of the RP model containing 12 molecular descriptors and molecular fingerprints is better than that of the model only with molecular descriptors. Of all the molecular fingerprints, ECFP_6 and FCFP_6 both have better classification effect in training set and test set. The corresponding highest *C* value of Matthews correlation coefficient was 0.717 and 0.733, respectively (see figure S1). Based on molecular descriptors and ECFP_6, the sensitivity and specificity of RP model were 0.848 and 0.852, respectively. The prediction accuracy of inhibitors and noninhibitors was 70.3% and 90.4%, respectively.

**Figure 2:**
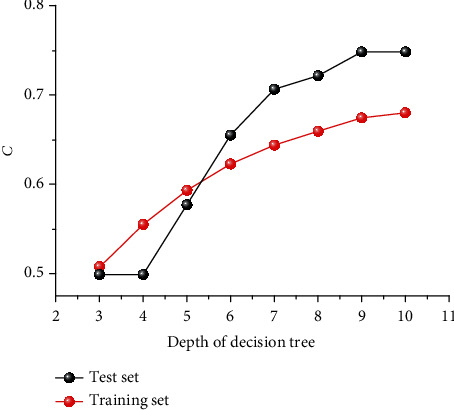
The *C* value changes of the training and test sets along with tree depth.

In RP model, depth is an important parameter determining the complexity of decision tree. Generally, the larger the tree depth, the more accurate the recognition of important features in the training set, but it also increases the risk of overfitting; the smaller the tree depth, the higher the tree applicability to datasets.  Figure 2 shows the *C* value changes of the training and test sets along with tree depth, which are used to evaluate the response ability of the models. From the training set, the *C* value increases with the growing of tree depth. For the test set, the *C* value reaches a maximum of 0.748, when the tree depth is 9. In order to avoid overfitting phenomenon, setting the tree depth of RP model to 9 is the best choice.

Table S1 lists the decision tree reports with tree depth of 9. In the mixed matrix, experimental data and prediction results are filled in the vertical and horizontal columns, respectively, where 0 and 1 represent HIV-1 IN inhibitor and noninhibitor, respectively. Based on the above equations (8) and (9), the prediction accuracies of inhibitors and noninhibitors are 0.825 and 0.862, respectively, with Matthews correlation coefficient of 0.722.

**Figure 3:**
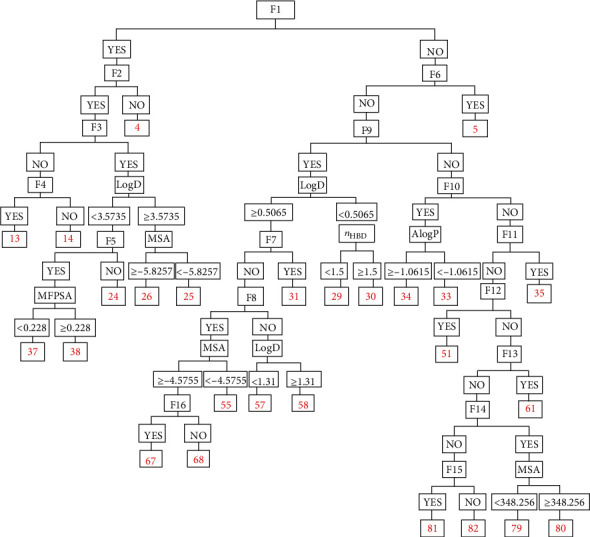
Decision tree with a depth of 9.

Figure 3 shows all the details of a decision tree with a depth of 9. It can be seen that the decision tree has 25 internal nodes and 26 leaves. The discriminant descriptors consist of 7 molecular properties (ECFP_6, logD, MSA, AlogP, *n*_HBD_, MFPSA, and MW) and 16 structural fragments (F1 … F16). These 16 molecular fingerprints are helpful to distinguish inhibitors from noninhibitors and have positive reference value to the following drug design (see figure S2).

### 3.3. Classification Based on Naive Bayesian Model

Although the decision tree obtained by RP model is concise and explicit, this method is highly sensitive to predetermined parameters and it will lead to false positives (FP) and false negatives (FN). To further improve model accuracy and make comparison, we used naive Bayesian (NB) method to establish another new classification model. The process of NB classification is to find features with separation ability in an unbiased way, which does not involve parameter fitting and adjustment as an unsupervised learning method.

Table S2 shows the influence of different parameter combinations in the test and training sets on NB classification. As for the model only based on molecular descriptors, the sensitivity, specificity, and *C* value of training set were 66.9%, 71.9% and 0.561; while those of test set were 60.5%, 78.4%, and 0.57, respectively. Considering that Matthews coefficient *C* is unsatisfactory, several other types of NB models have also been constructed by combining molecular fingerprint and molecular descriptor, and the classification performance is significantly improved.

Meanwhile, ECFP_6 was selected to compare the prediction performances between RP and NB models.  Table 1 shows the cross validation of NB classification, from which the TP, FN, FP, and TN values of NB model are 627, 55, 210, and 893, respectively. It turns out that the prediction accuracy of inhibitors is 0.883, and that of noninhibitors is 0.872; the correlation coefficient *C* value of NB model is 0.852, which is slightly higher than RP model (see Table S1), indicating that the prediction ability of NB model is better.

**Figure 4:**
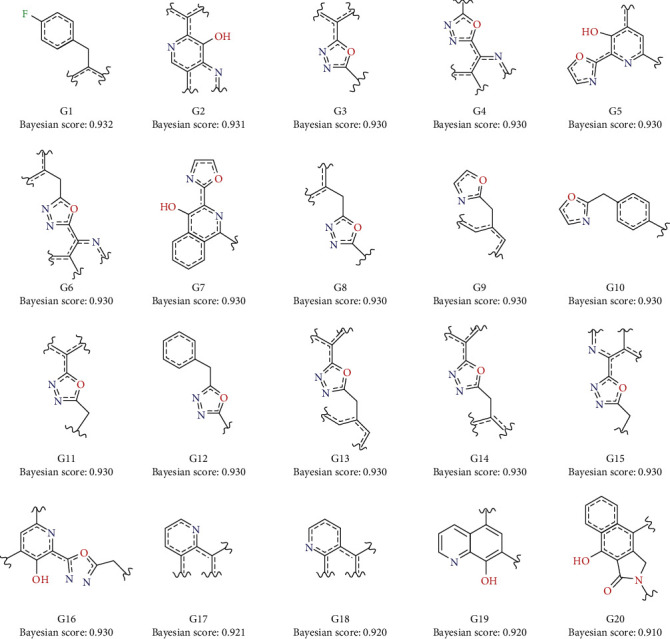
Potentially advantageous molecular fingerprint structures for HIV-1 IN inhibitors derived from naive Bayesian classification.

Like RP model, NB classification can also provide the unique key fragment structure (i.e., molecular fingerprints) of certain compounds. Molecular fingerprints can be transformed into two-dimensional fragments, which aids the design of HIV-1 IN inhibitors.  Figure 4 shows the top 20 potential favorable molecular fingerprints of HIV-1 IN inhibitors obtained by NB classification. Most of the advantageous fragments contain nitrogen-oxygen heterocycles (such as oxazole rings and pyridine rings), providing implications for molecular design based on inhibition mechanism and ligand structure. In addition, oxygen-sulfur double bond, nitrogen-nitrogen double bond, and pyrimidine ring appear in the unfavorable fragments (see Figures S3), which should be filtered out to improve the screening efficiency of HIV-1 IN inhibitors.

### 3.4. Molecular Design for Quinolinone Acid Inhibitors

**Figure 5:**
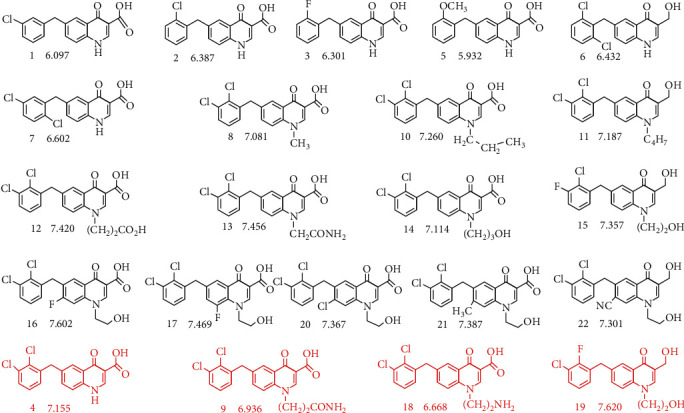
Structures and pIC_50_ values of quinolinone acid inhibitors against HIV-1 IN. The training set and test set are shown in black and red, respectively. The IC_50_ value is units of *μ*M.

Figure 5 shows structures and pIC_50_ of 22 quinolinone acid inhibitors, where 18 inhibitors were randomly selected into the training set to establish three-dimensional quantitative structure activity relationship (3D-QSAR) model. In the preprocessing step of 3D-QSAR analysis, the conformations of quinolinone acid inhibitors overlap quite well (see figure S4), which lays the foundation for the subsequent establishment of a good model.

**Figure 6:**
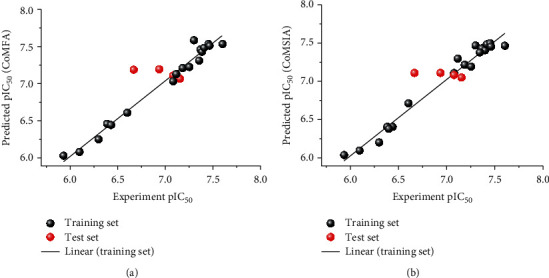
The correlation between experimental pIC_50_ and the predicted values by (a) CoMFA and (b) CoMSIA models.

As for the CoMFA models (see Table S3), the cross-validated correlation coefficient (*q*^2^) and noncross-validated correlation coefficient (*R*^2^) were 0.864 and 0.969, respectively. The root mean square error (RMSE) was 0.020, and the combination with high predicted correlation coefficient (*r*^2^_*p*_ = 0.918) confirms the reasonability and reliability of this model. According to the CoMFA model, the contribution rates of steric field (S) and electrostatic file (E) are 68.6% and 31.4%, respectively. It indicates that S has an important influence on the inhibitory activity of quinolinone acid inhibitors. In CoMSIA model, the contribution rates of S, E, H, D, and A were 7.4%, 13.2%, 16.9%, 45.7%, and 16.8%, respectively, which shows that the H-bond donors of quinolinone acid inhibitors have great influence on their activity. Then, the trained CoMFA and CoMSIA models both are used to predict molecular activity in the test set.  Figure 6 shows the correlation between experimental pIC_50_ and the predicted values by two models. It can be found that the correlation coefficient *R*^2^ was greater than 0.9, and the deviation between the predicted and experimental data was less than 1, which proves the reliability of the two models. In addition, some individual results are very consistent with their experimental data, such as compounds 4, 7, 10, and 14 in CoMFA model as well as compounds 4 and 10 in CoMSIA model.

It has been mentioned above that the two models complement and verify each other, which provides an important idea for the design of HIV-1 IN inhibitors. Figure S5 shows the CoMFA and CoMSIA contour map of quinolinone acid inhibitors, where compound 2 is used as the template and the contour line truncation is 80%: 20%. In the S field, there are large yellow and green blocks around the inhibitor, indicating that the introduction of large volume groups in the corresponding region is not conducive to and conducive to the enhancement of inhibitory activity. The yellow blocks are widely distributed around the ketone, carboxyl, and chlorine atoms; the green blocks are clustered near the nitrogen atom; it partly confirms why compounds 10 and 11 have better inhibitory activities. In the E field, the blue area near the ketone group indicates the introduction of a positively charged groups is conducive to improving inhibitory activity; there is a red block near the amino group, so the introduction of negatively charged group should be fully considered in molecular design. H-bond is one of the most important nonbonding interactions between drug molecules and receptors, which is the key to the drug-target specific recognition and biological activity. In the H field, almost all the hydrophobic chains are surrounded by gray blocks, and the introduction of hydrophobic groups will reduce the inhibitory activity. As for the D field, cyan represents the donor region of H-bond, where introducing hydroxyl or carboxyl group helps to improve inhibitory activity. In the A field of CoMSIA contour map, the red blocks near the carboxylic acid group and the contralateral nitrogen atom are mainly H-bond receptor rejection regions, and the introduction of H-bond receptors is strictly prohibited.

Based on the above analyses and previous studies, several design suggestions on improving the activity of quinolinone acid inhibitors are proposed: (1) the structure of beta-carbonyl carboxylic acid is strictly preserved, which is the key to maintain its activity; (2) at the N atom of amino group, long fatty chains (especially negatively charged one, such as carboxy group) are recommended; (3) H-bond donors (such as amino or hydroxyl group) may be considered for addition to the diphenylmethane side of quinolinone. To be objective, molecular dynamics (MD) simulation and biochemical enzyme experiments both are still needed to further verify the above molecular design ideas.

## 4. Conclusion

A database of HIV-1 IN inhibitors containing 1785 molecular structure and biological activity data was established first. The relationship between molecular descriptors and their inhibitory activities was systematically studied through the RP and NB methods. The prediction performance of the two classification models based on the combination of molecular descriptor with molecular fingerprint than that based on the individual molecular descriptor. By analyzing the key fragments transformed from molecular fingerprints, the nitrogen-containing ring including oxazole and pyridine rings is suggested to be introduced into subsequent inhibitor modification.

Finally, CoMSIA and CoMFA models with good predictive ability (*R*^2^ > 0.9) both were established by selecting quinolinone acid inhibitors against HIV-1 IN. According to the contour maps and the favorable groups given by NB classification, several design suggestions on improving the activity of inhibitors are propose. In particular, it is recommended to introduce long fatty chains (especially negatively charged one, such as carboxy group) into the N atom of amino group, as well as H-bond donors (such as amino group, hydroxyl group, and nitrogen-oxygen heterocycles) into the diphenylmethane side of quinolinone. This work provides some theoretical guidance for classification and molecular design of HIV-1 IN inhibitors.

## Figures and Tables

**Table 1:** Cross validation of naive Bayesian classification.

Model name	ROC score	ROC rating	TP	FN	FP	TN	SE	SP	*C*
Naive Bayesian model	0.897	Good	627	55	210	893	0.919	0.81	0.852

## Data Availability

The data used to support the findings of this study are available from the corresponding author upon request.

## Supplementary Materials

Supplementary Materials Figure S1: the *C* values of decision trees based on molecular descriptors and molecular fingerprints in the (a) training set and (b) test set. Figure S2: the 16 dominant fingerprints obtained from decision tree with depth of 9. Figure S3: potentially disadvantageous molecular fingerprint structures for HIV-1 IN inhibitors derived from naive Bayesian classification. Figure S4: structural superimposition of quinolinone acid inhibitors in the training set. Figure S5: CoMFA and CoMSIA contour maps of quinolinone acid inhibitors: (a) steric field, (b) electrostatic field, (c) hydrophobic field, (d) H-bond donor field, and (e) H-bond acceptor field. Table S1: decision tree report with tree depth of 9. Table S2: effects of different parameter combinations in the test set and training sets on naive Bayesian classification. Table S3: statistical parameters of CoMFA and CoMSIA models.
