# Supplementary material for: Classification and Design of HIV-1 Integrase Inhibitors Based on Machine Learning
Source: Comput Math Methods Med. 2021 Apr 1;2021:5559338. doi: 10.1155/2021/5559338 (PMC8035010; doi:10.1155/2021/5559338)
Supplement: Supplementary Materials — Figure S1: the C values of decision trees based on molecular descriptors and molecular fingerprints in the (a) training set and (b) test set. Figure S2: the 16 dominant fingerprints obtained from decision tree with depth of 9. Figure S3: potentially disadvantageous molecular fingerprint structures for HIV-1 IN inhibitors derived from naive Bayesian classification. Figure S4: structural superimposition of quinolinone acid inhibitors in the training set. Figure S5: CoMFA and CoMSIA contour maps of quinolinone acid inhibitors: (a) steric field, (b) electrostatic field, (c) hydrophobic field, (d) H-bond donor field, and (e) H-bond acceptor field. Table S1: decision tree report with tree depth of 9. Table S2: effects of different parameter combinations in the test set and training sets on naive Bayesian classification. Table S3: statistical parameters of CoMFA and CoMSIA models. [file 5559338.f1.docx]

**Supporting Information:**

**Classification and Design of HIV-1 Integrase Inhibitors Based on Machine Learning**

Junlin Zhou ^a, †^, Juan Hao ^a, †^, Lianxin Peng ^a, †^, Huaichuan Duan^a^, Qing Luo^a^, Hailian Yan ^a^, Hua Wan ^b^, Yichen Hu ^a^, Li Liang ^a^, Zhenjian Xie ^a^, Wei Liu ^a, *^, Gang Zhao ^a, *^, Jianping Hu ^a, *^

*^a^ Key Laboratory of Coarse Cereal Processing, Ministry of Agriculture and Rural Affairs, School of Pharmacy, Sichuan Industrial Institute of Antibiotics, Chengdu University, Chengdu 610106, China; ^b^ College of Mathematics and Informatics, South China Agricultural University, Guangzhou 510106, China*

^*^ Address correspondence to this author at the School of Pharmacy, Chengdu University, Chengdu 610106, China; Tel/Fax: 028-84616301; E-mails: [liuweicdu@sina.com](mailto:liuweicdu@sina.com), [zhaogang@cdu.edu.cn](mailto:zhaogang@cdu.edu.cn) and hjpcdu[@163.com](mailto:@163.com)

^†^ These authors contributed equally to this work.


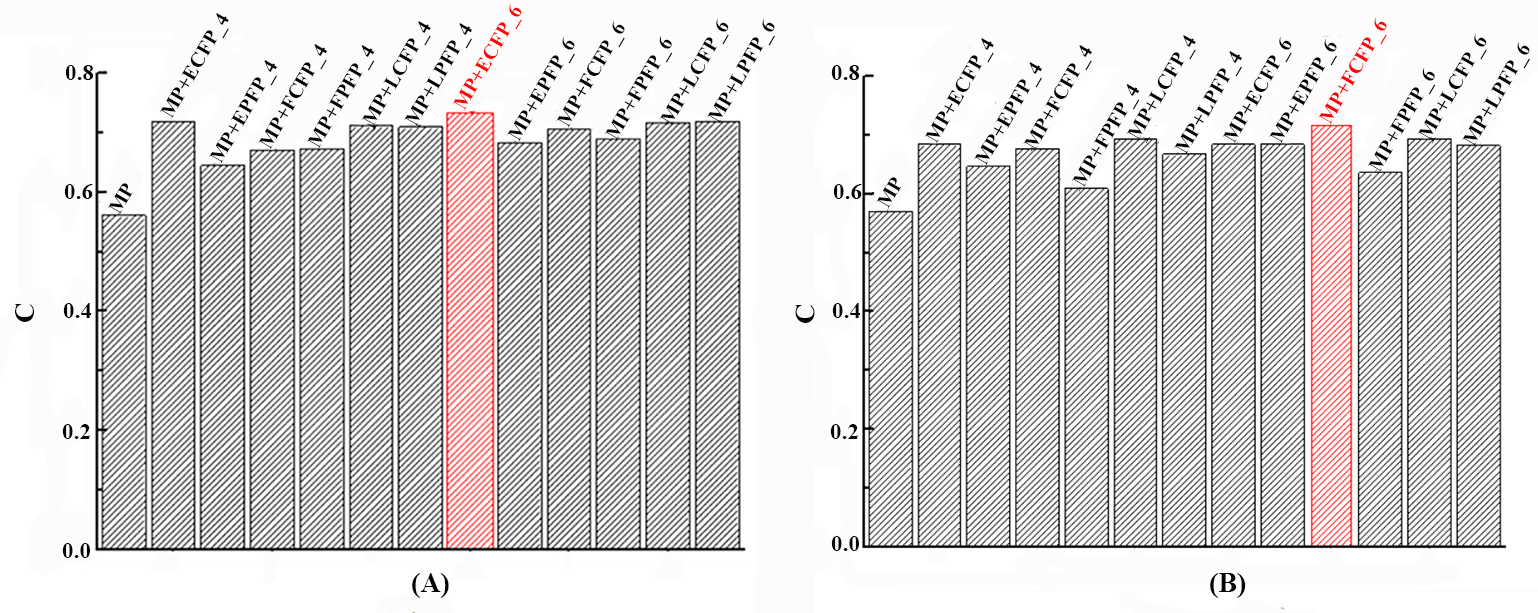


**Figure S1.** The C values of decision trees based on molecular descriptors and molecular fingerprints in the training set（A）and test set（B）


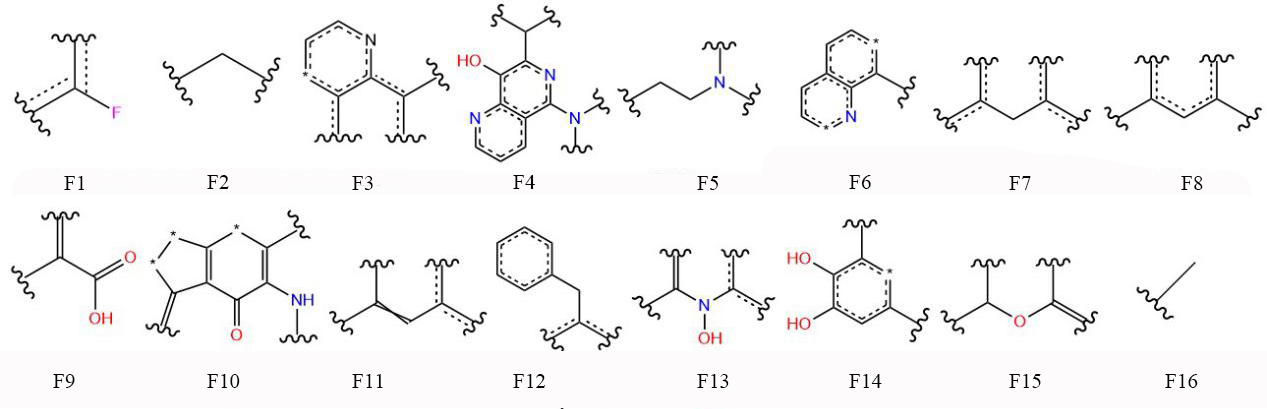


**Figure S2.** The 16 dominant fingerprints obtained from decision tree with depth of 9


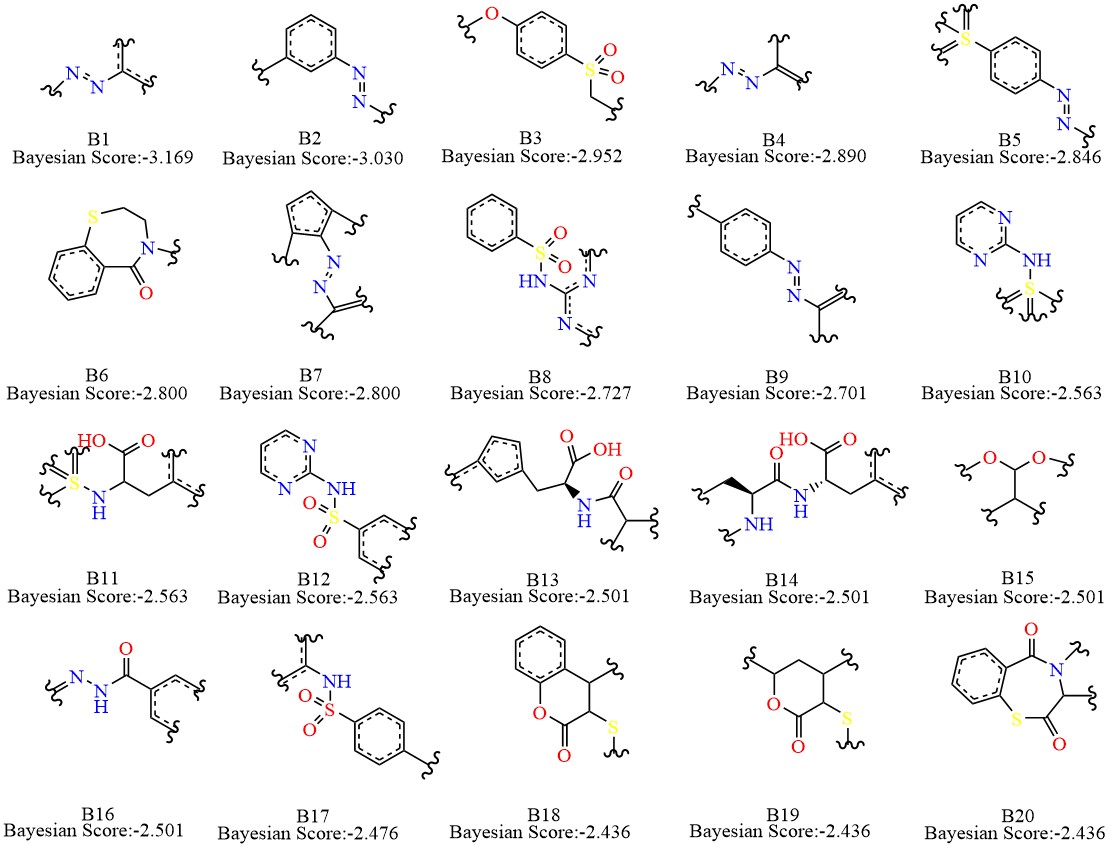


**Figure S3.** Potentially disadvantageous molecular fingerprint structures for HIV-1 IN inhibitors derived from naive Bayesian classification


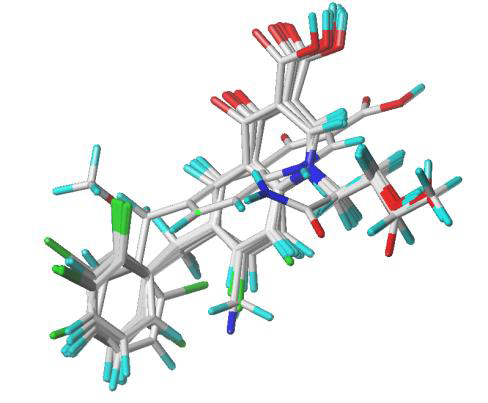


**Figure S4.** Structural superimposition of quinolinone acid inhibitors in the training set.


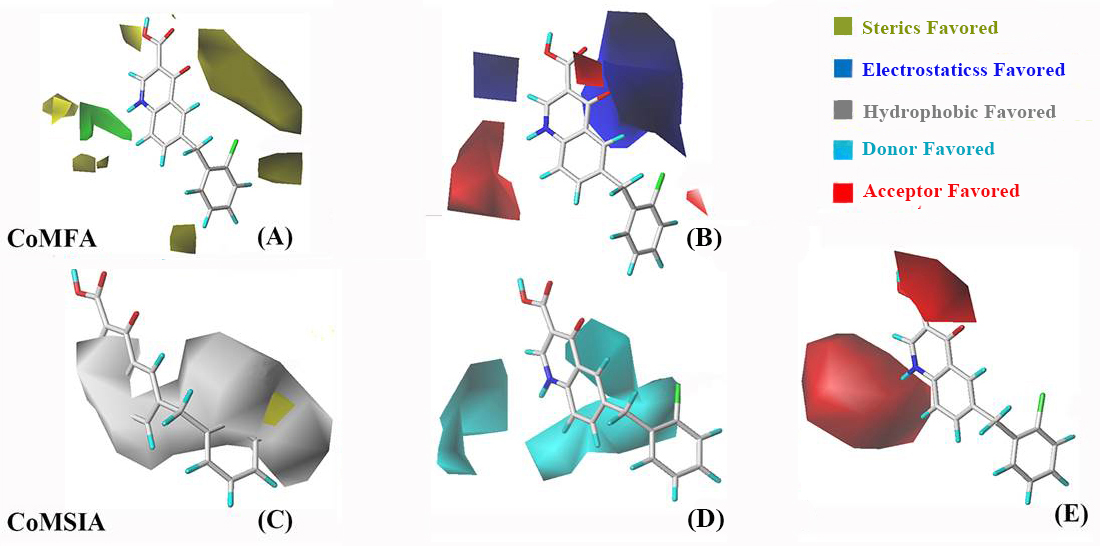


**Figure S5.** CoMFA and CoMSIA contour maps of quinolinone acid inhibitors:（A）Steric field, （B）Electrostatic field,（C）Hydrophobic field,（D）H-bond donor field,（E）H-bond acceptor field

**Table S1.** Decision tree report with tree depth of 9

| **Model information** | **Y axis properties** | **Mixed matrix** | **ROC score** | **Cross validation ROC score** |
| --- | --- | --- | --- | --- |
| tree:26 leaves  minimum value：0 | Class | \|  \| 1 \| 0 \| \| --- \| --- \| --- \| \| 1 \| 419 \| 134 \| \| 0 \| 89 \| 843 \| | 0.8578 | 0.8346 |

**Table S2.** Effects of different parameter combinations in the test set and training sets on naive Bayesian classification

| Descriptors | Test set | | | | | | | | | Training set | | | | | | | | |
| --- | --- | --- | --- | --- | --- | --- | --- | --- | --- | --- | --- | --- | --- | --- | --- | --- | --- | --- |
|  | TP | FN | FP | TN | SE | SP | Q_+_ | Q_-_ | C | TP | FN | FP | TN | SE | SP | Q_+_ | Q_-_ | C |
| MP | 78 | 51 | 37 | 134 | 0.605 | 0.784 | 0.678 | 0.724 | 0.570 | 370 | 183 | 262 | 670 | 0.669 | 0.719 | 0.585 | 0.785 | 0.561 |
| MP+ECFP4 | 98 | 31 | 26 | 145 | 0.760 | 0.848 | 0.790 | 0.824 | 0.684 | 467 | 86 | 152 | 780 | 0.844 | 0.837 | 0.754 | 0.901 | 0.718 |
| MP+EPFP4 | 86 | 43 | 21 | 150 | 0.667 | 0.877 | 0.804 | 0.777 | 0.647 | 423 | 130 | 190 | 742 | 0.765 | 0.796 | 0.690 | 0.851 | 0.645 |
| MP+FCFP4 | 94 | 35 | 23 | 148 | 0.729 | 0.865 | 0.803 | 0.809 | 0.677 | 475 | 78 | 217 | 715 | 0.859 | 0.767 | 0.686 | 0.902 | 0.670 |
| MP+FPFP4 | 88 | 41 | 36 | 135 | 0.682 | 0.789 | 0.710 | 0.767 | 0.609 | 390 | 163 | 111 | 851 | 0.705 | 0.885 | 0.778 | 0.839 | 0.673 |
| MP+LCFP4 | 88 | 41 | 11 | 160 | 0.682 | 0.936 | 0.889 | 0.796 | 0.694 | 481 | 72 | 176 | 756 | 0.870 | 0.811 | 0.732 | 0.913 | 0.711 |
| MP+LPFP4 | 91 | 38 | 22 | 149 | 0.705 | 0.871 | 0.805 | 0.797 | 0.667 | 478 | 75 | 174 | 758 | 0.864 | 0.813 | 0.733 | 0.910 | 0.709 |
| MP+ECFP6 | 97 | 32 | 25 | 146 | 0.752 | 0.854 | 0.795 | 0.820 | 0.684 | 469 | 84 | 138 | 794 | 0.848 | 0.852 | 0.773 | 0.904 | 0.733 |
| MP+EPFP6 | 97 | 32 | 25 | 146 | 0.752 | 0.854 | 0.795 | 0.820 | 0.684 | 431 | 122 | 150 | 782 | 0.779 | 0.839 | 0.742 | 0.865 | 0.683 |
| MP+FCFP6 | 98 | 31 | 18 | 153 | 0.760 | 0.895 | 0.845 | 0.832 | 0.717 | 480 | 73 | 180 | 752 | 0.868 | 0.807 | 0.727 | 0.912 | 0.706 |
| MP+FPFP6 | 95 | 34 | 36 | 135 | 0.736 | 0.789 | 0.725 | 0.799 | 0.636 | 434 | 119 | 146 | 786 | 0.785 | 0.843 | 0.748 | 0.869 | 0.689 |
| MP+LCFP6 | 99 | 30 | 25 | 146 | 0.767 | 0.854 | 0.798 | 0.830 | 0.693 | 488 | 65 | 178 | 754 | 0.882 | 0.809 | 0.733 | 0.921 | 0.716 |
| MP+LPFP6 | 91 | 38 | 18 | 153 | 0.705 | 0.895 | 0.835 | 0.801 | 0.682 | 471 | 82 | 156 | 776 | 0.852 | 0.833 | 0.751 | 0.904 | 0.718 |

**Table S3.** Statistical parameters of CoMFA and CoMSIA models

| **Models** | **PLS statistics** | | | | | | | | |  | **Contributions** | | | | |
| --- | --- | --- | --- | --- | --- | --- | --- | --- | --- | --- | --- | --- | --- | --- | --- |
|  | *ONC^a^* | | *q*^2 b^ | *E*_s_^c^ | *r*^2d^ | *F*^e^ | RMSE^f^ | *P*^g^ | *r*^2^_p_^h^ |  | *S*^i^ | *E*^j^ | *H*^k^ | *D*^l^ | *A*^m^ |
| **CoMFA** | 3 | 0.864 | | 0.104 | 0.969 | 145.234 | 0.020 | 0.000 | 0.918 |  | 0.686 | 0.314 | **-** | **-** | **-** |
| **CoMSIA** | 2 | 0.908 | | 0.110 | 0.963 | 195.530 | 0.016 | 0.000 | 0.933 |  | 0.074 | 0.132 | 0.169 | 0.457 | 0.168 |

^a^Optimum number of components, ^b^Leave-one-out (LOO) cross-validated correlation coeﬃcient, ^c^Standard error of estimate, ^d^Non-cross-validated correlation coeﬃcient, ^e^F-Test value, ^f^Root mean squared error (RMSE), ^g^Probability of r^2^, ^h^Predicted correlation coeﬃcient for the test set, ^i^Steric ﬁeld, ^j^Electrostatic ﬁeld, ^k^Hydrophobic ﬁeld, ^l^H-bond donor ﬁeld, ^m^H-bond acceptor ﬁeld.
